# Predictors for the utilization of community support systems against intimate partner violence among married women living with HIV in southwestern Uganda—A cross sectional study

**DOI:** 10.1371/journal.pone.0298397

**Published:** 2024-02-14

**Authors:** Edward Muteesasira, Davis Akampumuza, Dismus Abaho, Lillian Nuwasasira, Edward Kumakech

**Affiliations:** 1 Department of Physiotherapy, Faculty of Medicine, Mbarara University of Science and Technology, Mbarara, Uganda; 2 Department of Nursing, Faculty of Medicine, Mbarara University of Science and Technology, Mbarara, Uganda; 3 Department of Pharmacy, Faculty of Medicine, Mbarara University of Science and Technology, Mbarara, Uganda; 4 Department of Nursing, Faculty of Nursing and Midwifery, Lira University, Lira, Uganda; University of Michigan, UNITED STATES

## Abstract

**Background:**

Intimate partner violence (IPV) disproportionately affects married women living with HIV (MWLHIV), resulting in undesirable human rights, socio-economic, mental, maternal, and child health consequences. **C**ommunity **S**upport systems **a**gainst **V**iolence (CoSaV) are widely available and promising public and voluntary resources for the prevention and mitigation of IPV but are poorly investigated. We set out to identify the predictors for the utilization of the CoSaV among the MWLHIV.

**Methods:**

This was a quantitative cross-sectional study conducted among 424 consecutively sampled MWLHIV attending the Antiretroviral Therapy (ART) clinic at Kabale Regional Referral Hospital in southwestern Uganda in April 2021. Using an interviewer-administered questionnaire, data were collected on the participant’s socio-demographic characteristics, exposure to IPV, awareness about the CoSaV, perceptions about the quality, accessibility and challenges in accessing the CoSaV and the utilization. Modified Poisson regression model was used to identify the predictors for the utilization of CoSaV using the Statistical Package for Social Sciences (SPSS) version 23.0.

**Results:**

The mean age of the 424 participants in the study was 39.5 ± 10.2 years. More than half of the participants 51.9% (220/424) reported exposure to any IPV. Utilization of any CoSaV was found to be above average at 58.3% among the participants. The formal support (police, local government leaders, health workers and counselors) were more frequently utilized compared to the informal support (family, relatives and friends). Utilization of any CoSaV was higher among the women who were aware of the CoSaV and also those who were exposed to violence. Accessibility was identified as an independent predictor for utilization of any CoSaV.

**Conclusions:**

Intimate partner violence (IPV) was prevalent among MWLHIV in southwestern Uganda. However, the utilization of any CoSaV was suboptimal. The formal CoSaV were more frequently utilized than the informal support systems. Accessibility was an independent predictor for utilization of any CoSaV. There is need to improve access in order to increase the utilization of the CoSaV and contribute to the attainment of sustainable development goal 5.2.1 and end violence against women.

## Background

Intimate partner violence (IPV), a form of violence against women. “Intimate partner violence refers to any behavior within an intimate relationship that causes physical, psychological or sexual harm to those in the relationship” [1]. Intimate partner violence may take the form of physical, sexual, psychological or emotional violence. The violence is often perpetrated by the intimate partners or the relatives of the intimate partners for and on behalf of the intimate partners.

Intimate partner violence is a global public health problem. Globally in 2018, about 27% (uncertainty interval 23–31%) of ever-partnered women of reproductive age 15–49 years experienced physical or sexual, or both, IPV in their lifetime, and about 13% (10–16%) experiencing it in the past year [2]. Both the lifetime and past year IPV prevalence for sub-Saharan African countries (SSA) of 19–55% and 9–43% respectively were among the highest in the world in 2018 [2]. Uganda with an estimated past year IPV prevalence of 26% (18–36%) in 2018 was among the 14 countries with the highest intimate partner violence in the past year [2, 3].

Women living with HIV (WLHIV) are at a greater risk for IPV, stigma and discrimination compared to the women without HIV disease [4–7]. In SSA, the overall lifetime prevalence of any IPV was estimated to range from 30.2–33.0% [8, 9]. Among married Ugandan women living with HIV (MWLHIV), the lifetime prevalence of any IPV of 48.4% [10] was much higher than the 30.2–33.0% lifetime prevalence for SSA [2, 9] and also the 44.2% Uganda national lifetime prevalence for any IPV in 2016 (Uganda Bureau of Statistics (UBOS) & ICF, 2017 [11]).

All forms of IPV carry the risk for human rights, socio-economic, physical and mental health consequences to the women. For MWLHIV in particular, IPV carries the additional risk for poor maternal and child health outcomes such as mother to child transmission of HIV, miscarriage, abortion, preterm birth, low birth weight, postpartum depression, less breastfeeding and infant mortality [12, 13]. The IPV incident can disrupt access to healthcare and antiretroviral therapy (ART) for the MWLHIV. The ART inconsistency increases the risk of vertical transmission of HIV from mother to child during pregnancy, childbirth, or breastfeeding. The stress and physical trauma associated with IPV may elevate the risk of miscarriage or induce abortion, premature birth and lower birth weights, mental health issues, including postpartum depression. These create barriers to breastfeeding, either due to physical injuries or mental health issues, which can impact the infant’s nutrition and overall health. The combination of these factors, along with potential direct harm to the infant during violent incidents, increases the risk of infant mortality among children born to the MWLHIV experiencing IPV. Further evidence from SSA indicate that IPV is a barrier to ART initiation, adherence, compliance with clinic appointments, and positive biological outcomes (higher CD4 counts and low viral load) among WLHIV [14, 15].

The high burden, maternal and child health consequences of IPV underscores the importance of community support systems against IPV (CoSaV) in high-risk settings where proven interventions are not widely available. Community support systems against violence (CoSaV) are community-based formal, informal, public, voluntary and professional structures, resources and persons for peaceful governance of individuals, families, groups and also for community delivery of services. In Uganda, East African and other SSA, the CoSaV include formal and informal community-based local government leadership structures also known as local councilors (LCs), counselors, community health extension workers, health workers, police, human right defenders, cultural leaders, religious leaders, civil society or non-governmental organizations (NGOs), and information, education and communication (IEC) workers [16]. Others are the formal safe shelters and social protection programs [16]. These CoSaV are widely available in the communities. Therefore, CoSaV are promising first line response structures for the prevention and mitigation of IPV.

Despite their wide availability and closeness to the community, CoSaV were not well investigated in previous IPV studies in SSA settings [17–21]. The scarcity of some of the formal sources of support such as health workers makes CoSaV potential first-line mitigation measure against IPV. These essentialities or even the beneficial attributes of CoSaV may just be plausible assumptions. While there isn’t any clearly defined ratio of resources to women who need them, the realities of CoSaV in terms of their accessibility, utilization, helpfulness or protection for the MWLHIV in under-resourced communities in SSA remain areas for clarification through further research.

Kabale district located in rural southwestern Uganda is international business center for people from Uganda, Rwanda, Burundi, and Democratic Republic of Congo (DRC) and hence harbors a high population of WLHIV and IPV estimated at 36.6% [22]. The 36.6% level of IPV among MWLHIV in Kabale district was higher than the 30.2% lifetime IPV prevalence for SSA. Kabale district therefore provides appropriate SSA setting for examining the CoSaV for MWLHIV. We set out to determine the level of utilization of the CoSaV among the MWLHIV, the associated factors and predictors. The research findings will contribute to the attainment of the sustainable development goal (SDG) 5.2.1 which is to end IPV against women by 2030 [23].

The study was underpinned by the feminist theory which posits that spousal coercive control is not random but a purposeful and systematic men’s strategy to control and dominate their female partners [24]. The feminist theory argue that societal norms and structures contribute to the unequal distribution of power between men and women, creating an environment where violence can occur. Feminist perspectives on IPV consider broader social and structural factors that contribute to violence within intimate relationships. This includes issues such as economic disparities, gender roles, and societal expectations that contribute to power differentials and, in turn, violence. Feminist theory criticizes the traditional responses to IPV, such as criminal justice interventions, arguing that they may not address the root causes and may sometimes re-victimize survivors. Therefore, a more comprehensive and holistic approach that considers the social and structural context are advantageous. Feminist theory also explains the cycle of violence and the learned behavior associated with it whereby exposure to violence in one’s family or community can contribute to the perpetuation of violence across relatives or even generations. The feminist perspectives emphasize the agency of survivors and the importance of giving them a voice in shaping responses to IPV. This includes recognizing the diverse ways in which survivors cope with and resist violence. Last but not least, the feminist theory advocates for prevention strategies that address the root causes of IPV, such as challenging gender norms and promoting social change. This may involve educational programs, community engagement, and policy initiatives. These constructs of the feminist theory informed the items in the data collection tool, and analysis.

## Methods

### Study design and setting

This was a quantitative cross-sectional facility-based study [25, 26]. It is an appropriate design for prevalence and predictor related questions of this study [25, 26].

The study setting was Kabale Regional Referral Hospital (KRRH) which is located in Kabale district in southwestern Uganda. The hospital is a 280-bed facility. The hospital conducts more than 12,000 inpatient admissions and more than 82,000 outpatient visits per year. The hospital serves a population of approximately 2 million people. It serves as the regional referral facility for many districts in southwestern Uganda namely Kanungu, Kisoro, and Rukungiri, some parts of Ntungamo, as well as patients from the neighboring countries of Rwanda and the Democratic Republic of Congo (DRC). Typical of many referral hospitals in sub-Saharan Africa, KRRH has a vibrant ART clinic which serves 6,295 people living with HIV by 2009. The use of KRRH as the study setting made it possible to recruit diverse categories of MWLHIV.

The study area (Kabale district) is located in southwestern Uganda at a distance of about 560 kilometers from the Uganda’s capital city, Kampala. The district is a densely populated highland area with a population of 331,335 of which 52.2% are female and 76.3% live in rural parts of the district [27]. The residents of the district are predominantly Bakiga ethnic group who are traditionally subsistence farmers. Monogamous family is the commonest family type with a few polygamous families. Extended family set up with grandparents and relatives of the male partners is common in the area. Alcohol consumption is a common habit among both men and women in the area. Within the family, conflicts between the husband and wife or wife and husband’s relatives (in-laws|) are common and often arise from allegations of extramarital affairs and land wrangles. Illiteracy level among women in the area is considerably high at 30.3% [11]. The HIV prevalence in the general population of Kabale district was estimated at 4.3% (95% CI 2.4–6.3) which is lower than the southwestern Uganda regional estimate of 8% (95% CI 4–13) [28].

### Study population and sample size

The study was conducted among MWLHIV attending antiretroviral therapy (ART) clinic at KRRH in southwestern Uganda. We choose to conduct the study amongst the MWLHIV because of the huge burden of HIV-related IPV against them. We operationally defined eligible MWLHIV as WLHIV who have ever lived in the same household or slept on the same bed with a male intimate partner in a recognized marriage relationship for at least 1 year even if they later got separated, divorced or widowed. Studying women who were in marriage relationships for at least 1 year allows for a longitudinal perspective, gaining of insights into the development of intimate partner violence across marriage duration, including changes in the frequency and severity of violence and its impact on the survivors. Including women with varied marital experiences allowed the study to shed light on IPV incidents and dynamics over different forms of marital relationships, including ongoing marriages, and post-separation/divorce periods. It also allowed for the study to shed light on how the occurrence of IPV impacted on separation, divorce, or widowhood or vice versa. Furthermore, including separated or divorced women allows for the exploration of the persistence or even escalation of the IPV after separation or divorce or its impact on the survivors.

The sample size for study was 424 MWLHIV from Kabale district in southwestern Uganda. The sample size was calculated using the Cochran (1963) formula for calculating sample size for single proportion outcome variable [29]. The Cochran formula is appropriate for sample size determination for cross sectional studies as it calculates the sample size needed to achieve a certain level of precision in estimating population parameters with a specified level of confidence [30]. Since there were no previous related studies on the level of utilization of the CoSaV among MWLHIV in the region, we assumed the highest possible proportion of women who have ever utilized the CoSaV which was the outcome of interest (p) of 50% (0.5). Therefore, q which was the proportion of the population who did not utilize the CoSaV (1- p) was (1–0.5) which also equals to 0.5. At chosen the precision **e** of 0.05 and z value from standard normal distribution corresponding with 95% confidence interval of 1.96, the sample size n was calculated to be 385 MWLHIV. Given the risk for nonresponse from the sensitivity of the subject matter (IPV) under investigation, we assumed a 10% non-response rate and conducted a 10% adjustment of the initial 385 sample size (385 x 1.1) to the final sample size of 424 MWLHIV.

### Sampling method and participants’ recruitment procedures

Using a consecutive consenting sample selection procedure, we approached potential participants as they enter to seek ART services at Kabale regional referral hospital. We explained to them the purpose of the study and requested for consent. Women who accepted to participate in the study signed the informed consent form and were administered the data collection tool. We approached 430 women including the six participants who were later found to be ineligible women to attain the required sample size of 424 participants giving a response rate of 100.0%. Given the study’s focus on the violence among the married women, we excluded the six women from the study because they were later found to be never married nor cohabited although in or ever been in dating relationships. Violence among people in non-cohabiting dating relationships also known as dating violence is currently not regarded as a form of violence against women nor IPV [31]. The procedure for obtaining consecutive consenting sample described above enabled participant selection and sampling to be completed within a short period [25].

### Data collection tool and procedure

The interviewer-administered questionnaire for this study was adapted from a previous study on IPV screening [32]. The questionnaire was validated during a pretesting in a similar population in southwestern Uganda setting of Mbarara district amongst thirty participants in the local language of the setting (Runyankore-Rukiga). The responses and feedback obtained from the pretesting of the questionnaire was used to adjust the questionnaire. Reliability index Cronbach alpha testing for the scale section of the tool yielded a reliability coefficient of r equals to 0.81 which indicated the tool had a good internal consistency. Adjustments were made to the tool based on the pretest findings to improve the reliability and validity before its use for data collection in the actual study. In the full study, completion of each questionnaire took 15–30 minutes. We administered the questionnaire in the local language of the study setting (Runyankore-Rukiga) to enable participation of women without formal education. There were three trained Research Assistants and each administered the questionnaire to an average of 45 participants per day. The whole data collection process took the 4 weeks of the month of April 2021. The interviewer administration of the questionnaire allowed the interviewers to establish rapport, maintain eye contact, listen actively and take note of the body language of the participants [33]. In addition to allowing for the participation of illiterate participants, it also allowed the data collectors to clarify questions and cross check for understanding of the responses from the participants before noting on the questionnaire [33].

The data were recorded with pen directly on the questionnaire. All the response options for items of the questionnaire were pre-coded with exception of a few open-ended ‘others specify’ options which were coded after the data collection.

To ensure the fidelity and reliability of the data collection process, a comprehensive training for the interviewers was devised. This training involved an orientation session to familiarize the interviewers with the research goals, objectives, and ethical considerations. The study protocol, including the structured questionnaire, was thoroughly reviewed to ensure a clear understanding of the purpose of each question and the expected responses. Ethical considerations, such as confidentiality, informed consent, and participant well-being, were emphasized during the training. The interviewers engaged in role-playing exercises to simulate various interview scenarios, allowing them to practice using the questionnaire, refine communication skills, and address potential challenges. Cultural sensitivity training was provided to ensure interviewers were aware of and respected local customs, norms, and potential sensitivities related to HIV, IPV, and healthcare-seeking behavior. Technical training covered the practical aspects of administering the structured questionnaire, including the use of the pre-coded response options. Consistency checks were implemented during the training to assess the interviewers’ understanding and application of the protocol. Supervisors (the first and second authors of this manuscript) were designated to oversee the data collection process, providing guidance and ensuring adherence to the interview protocol. Fidelity to the interview protocol was further maintained through regular check-ins, supervisory oversight, and periodic reviews of completed interviews to assess the consistency of responses. Quality assurance measures, such as double-checking a sample of interviews, were implemented to enhance the reliability and validity of the collected data.

### Operational definitions and measures of variables

**Age** refers to the participant’s age in completed years at the time of recruitment and participation in the study. Age was verbally reported as a continuous data. For the purpose of sub group analysis for the age group most vulnerable to any IPV and age differences in IPV rates, age was categorized into distinct groups namely 18–35, 36–49 and 50+ years. These age groups represent reproductive younger, reproductive middle-aged, and older women respectively. These age categories reflect different life stages and transitions. Life experiences, relationship dynamics, and community support needs may vary significantly between younger, middle-aged, and older women. Categorizing by these age groups allows for the exploration of IPV prevalence and community support systems in the context of these life stages.

**Nature of relationship and level of contact with the partner** refers to the nature of the relationship and the level of contact with a partner among the participants. This was verbally reported as a categorical data as separated or divorced but still in occasional contact with the partner, living with or in regular contact with the partner, or widowed and not in any contact with any partner. The risk of IPV and the community support needs of women experiencing IPV can vary based on the nature of their relationship and contact with their partner. Women who are living with or in regular contact with a partner may face different risks and have different support needs compared to those who are separated or divorced but still in occasional contact. Categorizing based on these distinctions allows for understanding of IPV risk and support needs.

**Violence against women** refers to any act of gender-based violence that results in, or is likely to result in, physical, sexual or mental harm or suffering to women, including threats of such acts, coercion or arbitrary deprivation of liberty, whether occurring in public or in private life [31]. It includes different forms of violence against women and girls, such as intimate partner violence, non-partner sexual violence, trafficking, and harmful practices such as female genital mutilation. Violence against women can take the form of physical violence, sexual violence, and or emotional violence.

**Intimate partner** refers to a husband, cohabiting partner, boyfriend or lover, or ex-husband, ex-partner, ex-boyfriend or ex-lover [31].

**Intimate partner violence** refers to the behavior or action by an intimate partner that causes physical, sexual or psychological harm, including acts of physical aggression, sexual coercion, psychological abuse and controlling behaviors [31]. This definition covers violence by both current and former spouses and other intimate partners. Other terms used to refer to intimate partner violence include domestic violence, wife or spouse abuse, wife or spouse battering. Dating violence is usually used to refer to violence in intimate relationships among young people, which do not involve cohabiting.

**Support systems against intimate partner violence (CoSaV)** entails the provision of services such as police law enforcement and crime prevention, legal, housing and financial advice to the victims and perpetrators of violence [31]. It also entails facilitation of access to and use of community resources such as refuges or shelters, emergency housing, mediation by local leaders, psychological interventions and provision of advice on safety. Support system also includes advocacy, counselling, information, education and communication in confidence, in a safe and non-threatening environment.

**Counselling**, psychotherapy or a range of different psychological techniques provided by trained or untrained persons including family members, relatives and friends is another form of support system [31]. These approaches are provided in sex- or non-sex specific groups or couples, or on an individual basis. This can take many forms, one of the most common being therapies that are termed as cognitive behavioral therapies (CBT).

**Advocacy** is another form of support system which entails engaging with individual clients who are being abused or violated, with the aim of supporting and empowering them and linking them to community services [31]. In community settings, advocacy also entails bringing about systemic change, catalyzing increased recognition of women experiencing violence by various stakeholders. In this study, we define advocacy as support that entails the provision of legal, housing and financial advice; facilitation of access to and use of community resources such as police, local leaders, refuges or safe shelters, emergency housing, informal counselling, and provision of advice on safety.

**Safe shelter** also known as a safe house or refuge is another form of community support system for IPV [31]. It is usually a place, often at a secret or remote location from settlements, where women can flee from abusive partners. Safe shelters are usually run by a civil society or non-governmental organization (NGOs). It is a form of social and political response to IPV. It can also refer to a church or mosque, school facility, community hall, community group, association or other settings that provides a safe haven for women at risk or experiencing IPV.

**First-line support** refers to the primary psychological support, empathy and validation of IPV experiences that should be received by all women who disclose violence to formal and or informal service providers [31]. It shares many elements with what is being called “psychological first aid” in the context of crisis situations involving traumatic experiences.

#### Potential predictor variables

The variables on which data were collected included the participant’s socio-demographic characteristics such as age, marital status, monthly income and employment status. The participant’s socio-demographic characteristics were considered among the potential predictor variables for the study.

The participants were asked whether there were CoSaV they were aware of, the name, and the community issues they handle and the service providers. Also collected were data on the participant’s experiences of IPV following the disclosure of their HIV status to the male intimate partner and family members, the perpetrators and the allegations that instigated the IPV. Also collected were data on the participant’s perceptions of the quality, accessibility and challenges in accessing the CoSaV. The participant’s perceptions of the quality of the CoSaV were collected on a 4-point scale (poor, fair, good and very good). The participant’s perceptions of the accessibility of the CoSaV were collected on 3-point scale (easy, fair and hard).

Similarly, the participant’s perceptions of the challenges in accessing the CoSaV were collected on a yes or no question. The participants who indicated they perceived challenges in accessing the CoSaV were asked to specify the challenges.

**Experiences of IPV** refers to the women’s reported exposure to any form of violence within their current marital status from their intimate or non-intimate partners for and on behalf of their intimate partners. To measure the women’s experience of IPV, the women were asked whether they were ever violated physically, sexually or psychologically in their current marital status whether current married and living with the male intimate partner, separated, divorced or widowed or not? If violated, who violated them, whether the perpetrator was their male intimate partners or the non-intimate partners such as the relatives of the male intimate partner (in-laws)? And also if violated, what exactly triggered the violence, whether it was alcohol use (intoxication), accusations of infidelity, accusations of having multiple sexual partners, manifestation of symptoms of sexually transmitted diseases in the family members, inconsistent condom use or others such as land or business conflicts?

#### Outcome variable

The rate of utilization of the CoSaV by the MWLHIV was the outcome variable of the study. The participants were asked if they ever sought and utilized any CoSaV to mitigate the effects and or prevent the IPV, the specific intervention they received, and the providers of the CoSaV. The responses on the outcome variable (utilization of CoSaV) were dichotomized (1 = utilized the CoSaV or 0 = did not utilize the CoSaV) and treated as binary outcome variable in all statistical analysis.

### Data management and statistical analysis

The pre-coded questionnaire was used to further create the data entry template in EpiData version 3.1. Double data entry and validation with the source questionnaire were undertaken to minimize data entry errors [34]. The validated dataset in EpiData were exported to the Statistical Package for Social Sciences (SPSS) version 23.0 for windows for statistical analysis.

Frequency counts and percentages were calculated for all the variables. Descriptive statistics were used to calculate mean and standard deviations for the continuous data (age and income level) after confirming the normality of their distributions. The level of utilization of the CoSaV among the participants were calculated as frequency and percentage using the descriptive statistics. Multi-collinearity analysis using Variance Inflation Factor (VIF) for the two independent variables with numeral data i.e. the participant’s age and the level of income found an VIF value of <10 which showed no correlation between the two independent variables. For the rest of the categorical data (in this case age group, employment status, marital status, income level group, awareness about CoSaV, experiences of IPV and perceptions on CoSaV) were analyzed for their associations with the utilization of CoSaV using Chi square statistic and Kruskal-Wallis test [a non-parametric alternative to analysis of variance (ANOVA) to compare two or more groups].

Given the high (>10%) utilization rate for CoSaV among the participants, the modified Poisson regression with robust estimator was used to determine the main effect that is prevalence ratio (PR), the 95% confidence interval and the p values. All potential predictor variables associated with the outcome variable at p-values ≤0.2 from the bivariate analysis were entered into multivariate modified Poisson regression model [35]. The factors found to be associated with the outcome variable at p-value of ≤ 0.2 and entered into the multivariate analysis were the same surrogate markers for the constructs from the feminist theory of IPV such as gender norms, awareness of existing CoSaV, previous exposure to IPV, and agency of survivors. Consequently, the modified Poisson regression model fitted the utilization of CoSaV as the outcome variable and five potential predictor variables (nature of relationship and the level of contact with partner, exposure to IPV, awareness about CoSaV, perceptions of accessibility of CoSaV, and perceptions of the challenges in accessing the CoSaV). The model fitness was checked using Hosmer Lemoshew test at p-value of > 0.05 and was as desired non-significant. Crude prevalence risk ratio (CPRR), adjusted prevalence risk ratios (APRR) and their 95% Confidence Interval (CI) were calculated for the measures of association. Associations with p-value of ≤0.05 were considered to be statistically significant.

### Ethical considerations

The ethical approval for the study was obtained from the Mbarara University of Science and Technology Research Ethics Committee with the approval number MUSTREC 1/7. Potential participants were first screened for eligibility to participate in the study. None of the participants was aged below 18 years. Informed consent was obtained from all the participants aged 18 years and above. The participants were provided with information about the purpose of the study, methods of data collection, study time frame, risks, benefits, and freedom to refuse or withdraw participation from the study without penalties before enrollment into the study and data collection. The Research Assistants facilitated the informed consent process and the forms from the participants. Informed consents were obtained by written signature or thumb print. The participants were assured of confidentiality of the data during the study. The data collection tool was anonymized. Privacy was assured by conducting the data interviewer-administered questions in spaces with the hospital that were free from third party interference. Data on hard copy questionnaires were kept under lock and key only accessible to the research team. Electronic databases were password protected and the password were only made known to the research team.

## Results

### Participants’ socio-demographic characteristics

Four hundred and twenty-four (424) MWLHIV participated in the study, giving 100% response rate. Given their normal distributions, the participants’ mean age and monthly income were 39.5 ± 10.2 years and Uganda Shillings (UGX) 184,504 ± 265,289 (~US$ 53 ± 76) respectively. Over half, 61.8% (262/424) were living with and in regular contacts with their male intimate partners (Table 1).

**Table 1:** Socio-demographic characteristics of the married women living with HIV attending antiretroviral therapy clinic at Kabale regional referral hospital in southwestern Uganda (n = 424, April, 2021).

Variables	Description	Frequency (%)
**Mean age in years and the standard deviation**	39.5 ± 10.2 years	
**Nature of relationship and the level of contact with the partner**	Separated or divorced but still in occasional contact with the partner	92 (21.7%)
	Living with and in regular contact with the partner	263 (62.0%)
	Widowed and not in any contact with any partner	69 (16.3%)
**Mean monthly income in Ugandan Shillings (UGX) and the standard deviation**	UGX 184,504 ± 265,289 (US$ 53 ± 76)	
**Employment status**	Formal employment	51 (12.0%)
	Informal employment	307 (72.4%)
	Unemployed	66 (15.6%)

ART is Anti-Retroviral Therapy; UGX is Uganda Shillings; US$ is United States dollar; % is percentage.

### Reports of violence among the married women living with HIV

The findings indicate that more than half of the MWLHIV, 51.9% (220/424) were exposed to any form of violence. The 60.5% (159/263) of the experiences of violence among the women living with their male intimate partners was significantly higher than the 37.9% (61/161) experienced among the separated or divorced or widowed women in the study (*X*^2^ (1) = 19.480, p<0.0001). There were no age differences in the experiences of violence among the women (*X*^2^ (2) = 1.037, p = 0.595).

Most of the violence were found to be perpetrated by the male intimate partners 85.6% (185/216) compared to 14.4% (31/216) violence perpetrated by the non-intimate persons in this case the relatives of the male intimate partners.

The issues frequently reported to have triggered the violence included alcohol use [73, (33.8%)], accusations of partner infidelity [63, (29.2%)], accusation of having multiple sexual partners [44 (20.4%)], manifestations of symptoms of sexually transmitted infection in the family members [31, (14.4%)], inconsistent condom use [22, (10.2%)], and others such as land or business conflicts [21, (9.7%)].

### Awareness about the community support system against violence (CoSaV) among the married women living with HIV

The majority of the participants 82.5%, (350/424) demonstrated awareness of at least one of the CoSaV. The popular CoSaV among the participants were police law enforcement and crime prevention services (25.2%, 107/424), local government leaders (local councilors) mediation services (22.4%, 95 /424), health services (23.6%, 100/424), counseling (17.7%, 75/424), civil society or non-governmental organizations services (1.9%, 8/424), human rights defense (1.7%, 7/424), and information, education and communication services (1.4%, 6/424).

Awareness about the CoSaV were significantly higher among the women living with their male intimate partners compared to those who were separated, divorced or widowed (*X*^2^ (2) = 31.948, p< 0.0001). Similarly, awareness about the CoSaV were significantly higher among the women with higher income compared to their counterpart (*X*^2^ (2) = 23.750, p< 0.0001). However, there were no employment status differences in awareness about CoSaV among the participants.

### Perceptions regarding the quality of, accessibility to and challenges of accessing the CoSaV among the married women living with HIV

The findings indicate that about a fifth of the women (21.5%, 91/424) perceived the CoSaV as of poor quality. Only 11.1% (47/424) and 19.4% (82/424) of the women perceived the quality of the CoSaV as fair and good respectively.

Regarding perceptions about accessibility of the CoSaV, just a quarter of the women 25.7% (109/424) perceived CoSaV as fairly easy to access with only 24.8% (105/424) of them have perceived existence of challenges in accessing the CoSaV. There was no age, marital status, employment status and income level differences among the women regarding their perceptions about the accessibility and existence of challenges in accessing the CoSaV.

The perceived challenges in accessing the CoSaV included the high transportation costs (12.0%, 51/424), lack of time to pursue redress for IPV (6.1%, 26/424), inadequate information about human rights and procedures for pursuing redress for IPV (5.2%, 22/424), demand for money by the authorities in order to resolve the reported IPV cases (2.1%, 9/424), denial of permission to pursue redress for IPV cases by family members (2.1%, 9/424), and other challenges (1.9%, 8/424) such as the lack of special courts for hearing IPV cases, limited number of trained personnel to handle IPV cases, unfairness and injustices in IPV case handling, fear of disclose of HIV status to the wider community and the associated stigma and discrimination and the lack of penalties for perpetrators of IPV.

### Utilization of the CoSaV among the married women living with HIV

The overall level of utilization of the CoSaV among the MWLHIV was found to be 58.5% (248/424). The 69.1% (242/350) level of utilization of CoSaV among the women who demonstrated awareness of the CoSaV was found to be significantly higher than their counterpart (*X*^2^ (1) = 91.228, p< 0.0001). Similarly, the 99.5% (219/220) level of utilization of CoSaV found among the women who have experienced violence were significantly higher than that of their counterpart (*X*^2^ (1) = 313.931, p< 0.0001).

The most frequently utilized CoSaV were police law enforcement and crime prevention services 25.5% (108/424), the local government leaders’ mediation services 20.3% (86/424), health services 18.2% (77/424), counseling services 16.3% (69/424), information, education and communication services 3.1% (13/424), civil society or NGO advocacy services 1.2% (5/424) and human rights defense services 0.7% (3/424). Accordingly, the most frequently reported providers of the CoSaV were police 35.1% (87/248), nurses and doctors 29.8% (74/248), local government leaders 23.4% (58/248), counselors 17.7% (44/248), family members 3.6% (9/248), relatives and friends 2.4% (6/248), religious leaders 2.4% (6/248) and others such as community health extension workers 1.2% (3/248).

### Factors associated with the utilization of the community support system against violence among the married women living with HIV

The results from the bivariate analyses shown in Table 2 indicate that the factors that were significantly associated with utilization of the CoSaV among the MWLHIV were their marital status, awareness about the CoSaV, exposure to violence, perception of the accessibility to the CoSaV and the perceptions of challenges in accessing the CoSaV.

**Table 2:** Factors associated with the utilization of the community support systems against violence among the married women living with HIV attending the antiretroviral therapy clinic at Kabale regional referral hospital in Southwestern Uganda (n = 424, April 2021).

Factors		Utilization of the CoSaV		*Test for Association*
		Utilized F (%)	Not utilized F (%)	
**Age group in years**	18–35	99 (59.6)	67 (40.4)	*X*^2^ (2) = 0.150, p = 0.928^kw^
	36–49	107 (57.8)	78 (42.2)	
	50+	52 (57.5)	31 (42.5)	
**Monthly income level in Ugandan shillings**	Low income	171 (60.2)	113 (39.8)	*X*^2^ (2) = 2.587, p = 0.274^kw^
	Moderate income	44 (52.4)	40 (47.6)	
	Above moderate income	4 (80.0)	1 (20.0)	
**Nature of relationship and the level of contact with the partner**	Separated/ divorced but still in occasional contact with the partner	44 (47.8)	48 (52.2)	X^2^ (2) = 31.009, p< 0.0001^kw^*
	Living with and in regular contact with the partner	180 (68.4)	83 (31.6)	
	Widowed and not in any contact with any partner	24 (34.8)	45 (65.2)	
**Employment status**	Formal employment	35 (68.6)	16 (31.4)	X^2^ (2) = 2.461, p = 0.292^kw^
	Informal employment	175 (57.0)	132 (43.0)	
	Unemployed	38 (57.6)	28 (42.4)	
**Awareness about the CoSaV**	Aware	242 (69.1)	108 (30.9)	X^2^ (1) = 91.228, p< 0.0001*
	Unaware	6 (8.1)	68 (91.9)	
**Exposure to violence**	Violated	219 (99.5)	1 (0.5)	X^2^ (1) = 313.931, p< 0.0001^f^*
	Not violated	29 (14.2)	175 (85.8)	
**Perceptions of the quality of the CoSaV**	Poor	91 (100.0)	0 (0.0)	X^2^ (2) = 1.691, p = 0.429^kw^
	Fair	47 (100.0)	0 (0.0)	
	Good	81 (98.8)	1 (1.2)	
**Perception of the accessibility to the CoSaV**	Easy	81 (98.8)	1 (1.2)	X^2^ (2) = 11.657, p = 0.003^kw^*
	Fair	104 (95.4)	5 (4.6)	
	Hard	11 (78.6)	3 (21.4)	
**Perception of the challenges in accessing the CoSaV**	Yes	101 (96.2)	4 (3.8)	X^2^ (1) = 8.248, p = 0.004^f^*
	No	54 (81.8)	12 (18.2)	

x^2^ is chi square; df is degree of freedom; p is significance level, f is frequency, % is percentage; f is Fisher’s exact test; kw is Kruskal-Walli’s test and * is statistically significant.

### Predictors for the utilization of community support system against violence among the married women living with HIV

As shown in Table 3, from the multivariable modified Poisson regression model, the independent predictor for the utilization of the CoSaV among the MWLHIV was found to be the women’s perception of the accessibility of the CoSaV.

**Table 3:** Predictors for the utilization of CoSaV amongst the married women living with HIV attending the antiretroviral therapy clinic at Kabale regional referral hospital in Southwestern Uganda (n = 424, April 2021).

Factor		Crude PRR (95% CI)	Adjusted PRR (95% CI)	*P-* value for the Adjusted PRR (95% CI)
**Nature of relationship and the level of contact with the partner**	Separated or divorced but still in occasional contact with the partner	1.139 (0.979–1.326)	1.049 (0.940–1.170)	0.392
	Living with and in regular contact with the partner	1.400 (1.235–1.588)	1.059 (0.963–1.164)	0.238
	Widowed and not in contact with any partner	1.000	1.000	
**Awareness about the CoSaV**	Aware	1.841 (1.702–1.992)	1.398 (0.954–2.049)	1.398
	Unaware	1.000	1.000	
**Exposure to violence**	Violated	2.347 (2.236–2.465)	1.128 (0.988–1.288)	0.075
	Not violated	1.000	1.000	
**Perception of the accessibility of the CoSaV**	Easy	1.224 (0.886–1.519)	1.166 (1.014–1.342)	0.031*
	Fair	1.183 (0.951–1.472)	1.193(1.032–1.378)	0.017*
	Hard	1.000	1.000	
**Perception of the challenges in accessing the CoSaV**	Yes	1.155 (1.045–1.276)	1.015 (0.971–1.061)	0.507
	No	1.000	1.000	

PRR is Prevalence Ratio; CI is Confidence Interval; p is significance level and * is statistical significance p value.

## Discussion

The current study found more than half (58.5%) of the MWLHIV have utilized the CoSaV. This 58.5% level of utilization of the CoSaV found among MWLHIV from southwestern Uganda is almost double the 33% level of help-seeking for IPV reported among the general population of married women during the 2016 Uganda demographic health survey (UDHS) [10, 11]. Similarly, the 58.5% level of support-seeking for IPV found among MWLHIV in southwestern Uganda in currently is also higher than the 42% level of help-seeking for IPV found among WLHIV in Canada by Gormley et al (2022)- [36]. The higher level of utilization of CoSaV among the MWLHIV in southwestern Uganda in this study was associated to awareness about the CoSaV (x^2^ 91.228, df 1, p< 0.0001) and also to the perceived accessibility of the CoSaV (x^2^ 313.931, df 1, p< 0.0001). Besides awareness and accessibility, the higher level of utilization of the CoSaV among MWLHIV in southwestern Uganda found in this study is attributable to the current study methodology that examined for the women’s utilization of both formal and informal CoSaV which were not examined that comprehensively in the previous studies. Although higher than the levels reported in the previous studies, the 58.5% level of utilization of CoSaV found among MWLHIV in southwestern Uganda exposed to IPV still falls below the Sustainable Development Goal and target 5.2.1 of ensuring all (100%) of IPV cases reported, investigated and sentenced/ addressed [37]. The suboptimal utilization of CoSaV among MWLHIV call for efforts to further improve the utilization of formal and informal CoSaV to prevent and mitigate effects of IPV among women and girls.

Our study in southwestern Uganda found that the most utilized CoSaV among the MWLHIV experiencing IPV were law enforcement by the Police (25.5%), mediation by the local government leaders in particular the local councilors (20.3%), health services (18.2%) and counseling (16.3%). Our study finding that health services is among the most frequently utilized CoSaV among the MWLHIV experiencing IPV concurs with a study conducted among WLHIV in Canada by Gormley et al (2022) which also found health services as the most frequently utilized support system for IPV [36]. Conversely, the 16.3% level of utilization of counseling services for IPV found among the MWLHIV in our study in southwestern Uganda is about half of the 40.2% level of utilization of counseling services found in a previous study conducted among the WLHIV in southwestern Uganda by Arishaba et al (2022) [38]. The difference could be due to the differences in the study populations. The previous study in southwestern Uganda by Arishaba et al (2022) included never married women and hence considered help-seeking for dating violence which our study did not consider. Nonetheless, these studies indicated the growing use of the formal support systems in this case law enforcement by police, mediation services by the local leaders (local councilors), health services and counseling services over the informal support systems for IPV among MWLHIV experiencing violence. This finding about frequent use of formal support systems for IPV disagrees with the 2016 UDHS findings which showed that women’s most sought source of help for IPV were informal support systems such as women’s own family members, their partner’s relatives and friends [11]. The high women’s awareness about the role of the formal support systems such as police, local government leaders in particular local councilors, health workers and counselors for IPV compared to the informal support systems such as family members, relatives and friends may explain their frequent use. The current study findings that the formal support systems (such as police, local government leaders, health workers and counsellors) were the most utilized sources of support for IPV among the MWLHIV exposed to IPV in southwestern Uganda concurs with a previous study conducted among Korean victims of IPV that also found that women exposed to IPV contacted the formal support systems (such as police, nurses and doctors) with the exception of a few women of low income status who contacted the informal support systems (such as family, relatives and friends) for IPV [39].

In this study in southwestern Uganda, the law enforcement services, particularly the Police, were identified among the popular CoSaV among the MWLHIV. Law enforcement agencies play a crucial role in ensuring the protection and safety of people at risk of IPV [40]. This involves responding promptly to emergency calls related to IPV, issuing restraining orders when necessary, and providing legal protection to prevent further harm. Furthermore, law enforcement is instrumental in investigating and prosecuting cases of IPV. This includes conducting thorough investigations, gathering evidence against perpetrators, and collaborating with the legal system to ensure the prosecution of those responsible for IPV. Supportive services are another key aspect of law enforcement’s role. They collaborate with social service agencies to refer victims to counseling, shelters, and healthcare services. Law enforcement also advocates for the rights and needs of victims within the legal system, ensuring they have access to necessary services. Moreover, law enforcement agencies work in coordination with healthcare providers to ensure that victims have access to medical services, including injury wound care, HIV-related care and support. They also establish community policing centers, creating accessible spaces where individuals can report incidents, seek assistance, and access information in a safe and supportive environment.

The current study in southwestern Uganda indicated the significant independent predictor for the utilization of the community sources of support for IPV among the MWLHIV was the easiness in the accessibility of the community support services. This findings concurs with a previous study conducted in southwestern Uganda that the availability and hence accessibility of the sources of support such as counselor was a significant independent predicator of care-seeking for IPV among WLHIV [38]. These findings imply that the utilization of community sources of support for IPV among the MWLHIV may be improved by making the CoSaV easily accessible to the women.

### Study strengths and limitations

The fact that the study was conducted among diverse categories of MWLHIV points to the representativeness of the sample and the wide applicability of the findings. The assessment of the women’s utilization of both formal and informal forms of support for IPV including mediation services by the local leaders unlike previous studies is another strength of the study. Nonetheless, the study had some limitations. The participant recruitment and data collection were conducted in April 2021 during the national lockdown for COVID-19 pandemic which may have affected on the participation of some of the MWLHIV. This risk was mitigated by giving ample time (about 1 month) for the participant recruitment and data collection exercise which increased the time period for the eligible women to participate until the required sample size was attained. The COVID-19 lockdowns may have contributed to the high prevalence of IPV incidents observed among the MWLHIV in this study even though no data were collected on the IPV incidents before the COVID-19 lockdowns to compare with the incidents collected during the COVID-19 lockdowns for this study. Otherwise, other research also found increases in the frequency and severity of IPV during the COVID-19 pandemic [41–43]. Notably, data on IPV experiences by the specific forms of IPV (physical, sexual, and emotional) were not collected and consequently, the IPV incident, sub group analysis and modeling could not be performed according to these three forms of IPV in addition to the overall prevalence. The omission happened because the primary outcome of the study was the extent of utilization of the CoSaV following IPV incident and its predictors rather than an investigation into the IPV incidents, the types and their risk factors. The use of verbal report to establish whether the participants utilized the formal or informal sources of support for IPV carried the risk of recall bias. We minimized the risk for recall bias by administering the questionnaire by interview which provided for deeper probing, clarifying and hints to aid recall. Furthermore, the administration of the questionnaire by interview which provided for deeper probing and clarification minimized the risk for social desirability bias arising from the sensitivity of the subject matter of IPV. Notably, as a convenience sample was used in this study, generalizability of the findings to other MWLHIV may be limited.

## Conclusions

More than half of the of the MWLHIV in Kabale district southwestern Uganda have ever experienced any form of IPV. Utilization of any CoSaV was just above average among the MWLHIV. The formal support (police, local government leaders, health workers and counselors) were more frequently utilized compared to the informal support (family, relatives and friends). Utilization of any CoSaV was higher among the women who were aware of the CoSaV and also those who were exposed to violence. The independent predictor for utilization of any CoSaV was the perceived easiness of accessing the CoSaV. There is need to improve access to CoSaV in order to increase its utilization and contribute to the attainment of sustainable development goal 5.1.1 and end violence against women. There is also need for further research into the IPV rates, risk factors and predictors by the type of IPV (physical, sexual and emotional) among MWLHIV.

## Abbreviations

ART: Antiretroviral therapy
CI: Confidence Interval
CoSaV: Community Support System against Violence
COVID-19: Corona Viral Disease 2019
Df: Degree of freedom
DRC: Democratic Republic of Congo
HIV: Human Immunodeficiency Virus
IEC: Information, Education and Communication
IPV: Intimate Partner Violence
KRRH: Kabale Regional Referral Hospital
LCs: Local Councilors
MUSTREC: Mbarara University of Science and Technology Research Ethics Committee
MWLHIV: Married Women Living with HIV
NGO: Non-Governmental Organization
P: p-value or significance level
PRR: Prevalence Risk Ratio
SDG: Sustainable Development Goal
SPSS: Statistical Package for Social Sciences
UBOS: Uganda Bureau of Statistics
UDHS: Uganda Demographic Health Survey
UGX: Uganda Shillings
US$: United States Dollar
WLHIV: Women Living with HIV
*X* ^2^: Chi square
